# Solitary Fibrous Tumors of the Central Nervous System: A Retrospective Study From a National Referral Hospital in Mexico

**DOI:** 10.7759/cureus.104633

**Published:** 2026-03-03

**Authors:** Carlos J Mávita Corral, Flavio Hernández González, Kevin S Toache, Pedro A González Zavala, Marco A Rodriguez-Florido, Luis G Castellanos Pallares, Yareli B Melo Estévez

**Affiliations:** 1 Neurosurgery, Hospital de Especialidades, Centro Médico Nacional Siglo XXI, Instituto Mexicano del Seguro Social, Mexico City, MEX; 2 Pathology, Hospital de Especialidades, Centro Médico Nacional Siglo XXI, Instituto Mexicano del Seguro Social, Mexico City, MEX

**Keywords:** central nervous system tumor, hemangiopericytoma (hpc), nab2-stat6 fusion, solitary fibrous tumor (sft), stat6

## Abstract

Background

Solitary fibrous tumors (SFTs) are rare mesenchymal neoplasms characterized by NAB2::STAT6 fusion. Despite their widespread occurrence, they are infrequent. SFTs typically manifest in adulthood with non-specific clinical symptoms. Surgical intervention remains the primary treatment modality, and the extent of resection is a critical factor in disease management.

Objective

To delineate the clinical and demographic characteristics, treatment status, and outcomes of patients with SFT in a national referral center.

Materials and methods

An observational, descriptive, and retrospective study was conducted involving patients treated at the Neurosurgery Department of a referral hospital in Mexico from January 2019 to December 2024. Clinical records, imaging studies, and pathological reports were reviewed. Frequency distributions and proportions were calculated for qualitative variables, and measures of central tendency were employed for quantitative variables.

Results

Ten patients were identified. The mean age was 48 years, with female predominance (seven patients = 70% of the cases). Tumors were supratentorial in 60% of the cases (six cases), 20% were infratentorial (two cases), and 20% were located in the spinal canal (two cases). The clinical and imaging features were nonspecific. The primary complication was significant intraoperative hemorrhage.

Conclusions

Due to their rarity, these tumors may be overlooked in clinical practice. The low availability of diagnostic tests in many centers may contribute to the underestimation of the incidence of SFTs.

## Introduction

A solitary fibrous tumor (SFT) is a mesenchymal neoplasm [1] characterized by a genomic inversion at the 12q13 locus, which leads to the fusion of the NAB2 and STAT6 genes, with nuclear expression of the latter. Although SFTs can manifest in any anatomical location, they constitute less than 1% of primary intracranial tumors [2-5]. These tumors most commonly occur in adults between the fifth and seventh decades of life [6]. The mean age at presentation is approximately 55 years, with no sex predilection. Intracranial location corresponds to 80.6% of the cases, intraspinal in 13.4%, and unspecified in 6% of the cases [7]. The clinical manifestations of SFTs are nonspecific and are influenced by tumor location, mass effect, and increased intracranial pressure [8]. The differential diagnosis should consider extra-axial tumors, particularly meningiomas, as well as dural metastases, lymphomas, sarcoidosis, and gliosarcomas. On CT imaging, SFTs appear as lesions with irregular borders, a lack of calcifications, or adjacent hyperostosis [9]. On MRI, they are isointense on T1-weighted images, with high or mixed intensities on T2-weighted images, and exhibit variable contrast enhancement [10]. An apparent diffusion coefficient (ADC) value greater than 1.15 and a susceptibility-weighted imaging (SWI) value exceeding 1.0 have been suggested to differentiate SFTs from meningiomas [11]. Spectroscopy revealed lipid and myoinositol peaks [12].

Despite these findings, there is no definitive, reliable data to differentiate SFTs from other neoplasms, although certain features, such as lobulated borders, heterogeneous contrast uptake, and a fungoid appearance, are associated with a more aggressive progression [13,14]. Total gross resection remains the standard treatment. It has been posited that the extent of resection is directly correlated with the survival outcomes. As with any surgical intervention, a balance between preserving functionality and achieving an adequate resection must be maintained [15]. Radiosurgery represents an additional option within the therapeutic arsenal and has demonstrated a reasonable rate of local control [16,17]. SFTs exhibit a high propensity for recurrence and can occasionally metastasize, adversely affecting prognosis even in grade 1 tumors, underscoring the importance of diligent follow-up. The reported overall survival rates at three, five, and 10 years were 89.7%, 79.4%, and 76.6%, respectively. The extent of resection, particularly during the initial surgery, has been identified as the primary determinant of tumor control, with a local control rate of 84% at five years in those with achieved total gross resection compared to 38% in those with subtotal resection [8]. Currently, SFTs are considered relatively understudied neoplasms, with limited case series available in the literature and a paucity of studies focusing on the Mexican population. Therefore, this study aimed to provide a comprehensive overview of the clinical and demographic characteristics, treatment, and progression of patients with these tumors. A retrospective study of 10 cases with emphasis on outcomes such as gross total macroscopic resection, postoperative functional outcome, complications (such as hemorrhage), and mortality was conducted for this purpose.

## Materials and methods

This was an observational, descriptive, and retrospective study that included patients admitted to the Neurosurgery Department of the “Dr. Bernardo Sepúlveda Gutiérrez” Specialty Hospital from January 2019 to December 2024, diagnosed with SFT. Patients were selected from the pathology database. Diagnosis was established using the WHO 2021 classification of Central Nervous Tumors, and this classification was used for histopathological grading. Clinical records, imaging system data, and pathology files from hospital archives were meticulously reviewed. Inclusion criteria were age ≥18 years, possession of a clinical record, histopathological diagnosis of SFT confirmed with WHO 2021 criteria, availability of histopathology slides, and surgical treatment in the Neurosurgery Department of the hospital during the specified period. Exclusion criteria were age <18 years, lack of clinical or radiological data, incomplete criteria according to the WHO 2021 criteria, unavailable histopathological material, and discordant histopathological diagnosis in case of multiple surgical sampling. Frequency distributions and proportions were calculated for qualitative variables, and measures of central tendency were employed for quantitative variables. Statistical analysis was performed using IBM SPSS Statistics for Windows, Version 30 (Released 2024; IBM Corp., Armonk, New York, United States) software. Follow-up and imaging protocol were consultant-dependent but, in general, consisted of at least on a two-month MRI, and biannual MRI for one additional year. MRIs included T1, T2, fluid-attenuated inversion recovery (FLAIR), ADC, and diffusion-weighted imaging (DWI) sequences with contrast-enhanced phases, and were evaluated by the radiology department and authors (CJMC and PAGZ). Recurrence was defined as the new appearance or tumoral growth between two consecutive MRIs. Tumoral control was defined as the absence of tumoral growth in a 12-month follow-up. This study adhered to the Strengthening the Reporting of Observational Studies in Epidemiology (STROBE) guidelines for observational studies. Ethical approval was obtained from the Local Health Research Committee 3601, with approval number R-2024-3601-299.

## Results

During the study period, SFTs constituted less than 0.5% of the total samples of central nervous system lesions submitted by the neurosurgery department. Table 1 summarizes the primary clinical and demographic characteristics. The mean age at the time of the initial surgery was 48.6 years (range, 27-70 years). Of the 10 cases diagnosed during the study period, seven were female (70%), and three were male (30%). The most prevalent tumor location was supratentorial (60%, n = 6), followed by infratentorial (20%, n = 2) and spinal (20%, n = 2) locations. The interval from symptom onset to the first surgical intervention averaged 11.2 months, with a range of 0-60 months; excluding spinal lesions, this interval was 5.3 months. The clinical manifestations vary according to the tumor location. Headache was the most frequent symptom, occurring in 75% of intracranial cases (six patients), and all intradural spinal cases presented with pain and paresthesia. One supratentorial case exhibited generalized tonic-clonic seizures, whereas another exhibited neurological deterioration. The primary diagnostic suspicion was meningioma in 70% of cases (seven patients), followed by schwannoma in 20% of cases (two cases). In the remaining case, located in the anterior cranial base with extension to the nasal fossa, the differential diagnosis included an inverted nasal papilloma.

**Table 1:** Clinical and demographic characteristics. F: female, M: male, N/A: not applicable

Case	Sex	Age	Tumor Location	WHO Grade	Clinical Presentation	Main Symptoms	Symptom Duration	Initial Radiologic Diagnosis
1	M	34	Supratentorial - anterior falx	1	Executive dysfunction; mood changes, apathy, abulia	Headache	6 months	Meningioma
2	M	39	Supratentorial - posterior falx	2	Decreased visual acuity	Headache	6 months	Meningioma
3	F	64	Supratentorial - posterior falx	3	Balance disturbance	Headache	3 months	Meningioma
4	F	58	Anterior skull base and nasal cavity	1	Proptosis	Periocular pain, nasal fullness	18 months	Inverted nasal papilloma
5	M	27	Parasagittal, middle third	1	Seizures	Headache	3 months	Meningioma
6	F	70	Parasagittal, middle third	1	Decreased level of consciousness	N/A	4 months	Meningioma
7	F	44	Infratentorial cerebellopontine angle	1	Hydrocephalus, gait lateropulsion	Headache	6 months	Schwannoma
8	F	55	Tentorial	1	Postural instability	Headache	1 month	Meningioma
9	F	49	Intradural spinal (C7-T2)	2	Monoparesis of lower limb	Paresthesias, pain	9 months	Schwannoma
10	F	46	Intradural spinal (L4-L5)	1	Paraparesis	Paresthesias, pain	60 months	Meningioma

**Figure 1:**
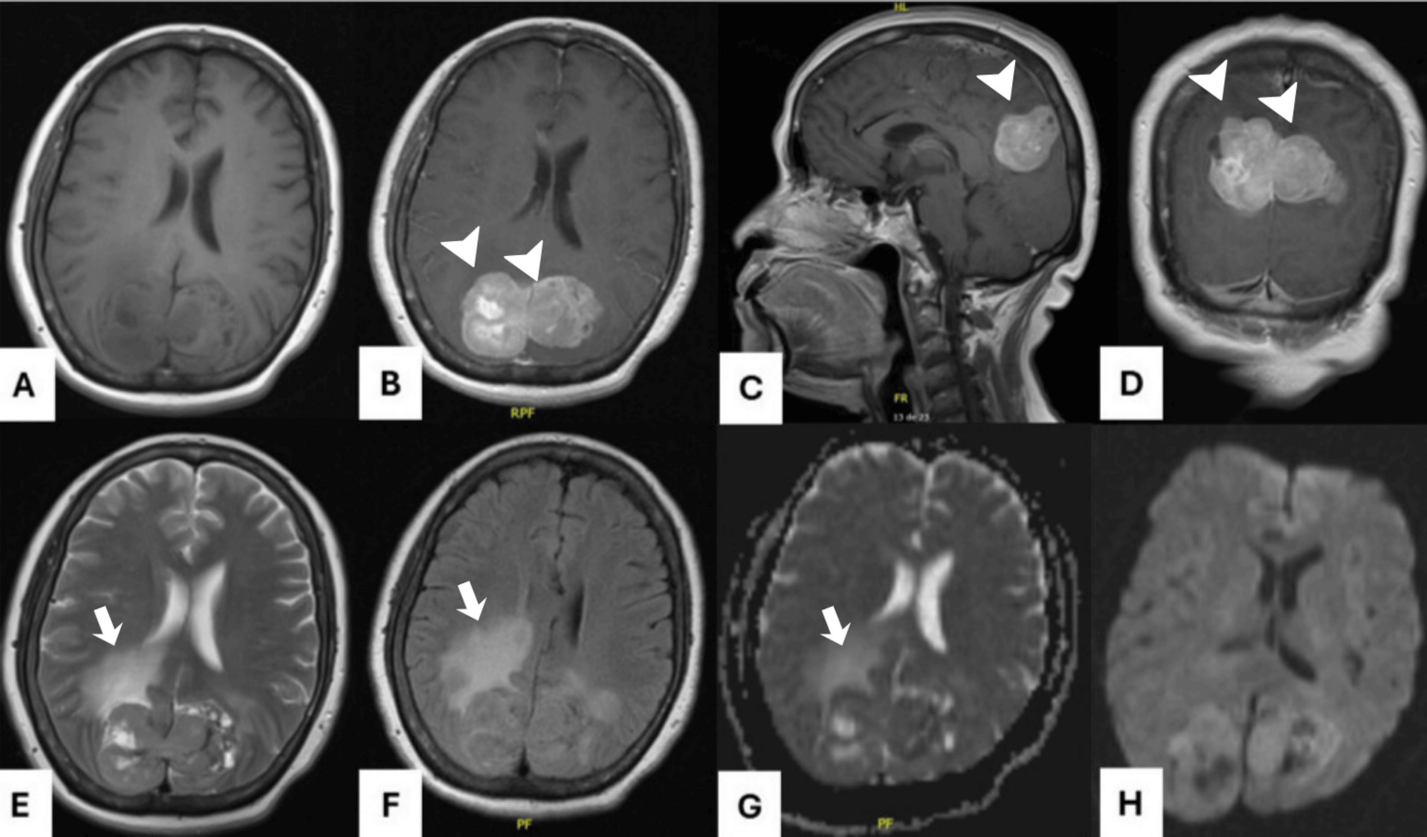
Bilateral lesion in the posterior third of the falx. The lesion appears isointense on (A) T1, (E) T2, and (F) FLAIR sequences, with a lobulated morphology and well-defined borders, presenting heterogeneous central areas. (B, C, D) Significant enhancement was observed after contrast administration. (G) On ADC, it appears predominantly hypointense, with some hyperintense areas. (H) The inverse of what is observed on DWI. Arrowheads: Enhancing lesion, Arrows: Peritumoral edema. FLAIR: fluid-attenuated inversion recovery; ADC: apparent diffusion coefficient; DWI: diffusion-weighted imaging

**Figure 2:**
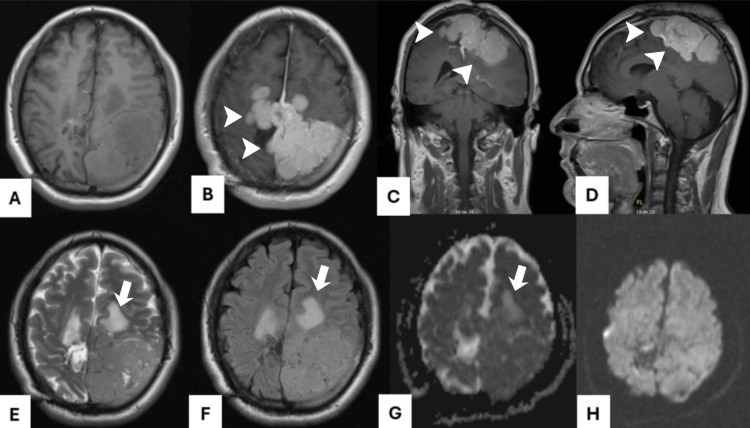
Predominant lesion in the middle third of falx. Tumor appears isointense on (A) T1, (E) T2, and (F) FLAIR, with a multilobulated morphology and well-defined borders, presenting small central heterogeneous areas. (B, C, D) Considerable enhancement was observed after contrast administration. (G) On ADC, it appears predominantly isointense (H) as in DWI. Arrowheads: Enhancing lesion. Arrows: Peritumoral edema. FLAIR: fluid-attenuated inversion recovery; ADC: apparent diffusion coefficient; DWI: diffusion-weighted imaging

**Figure 3:**
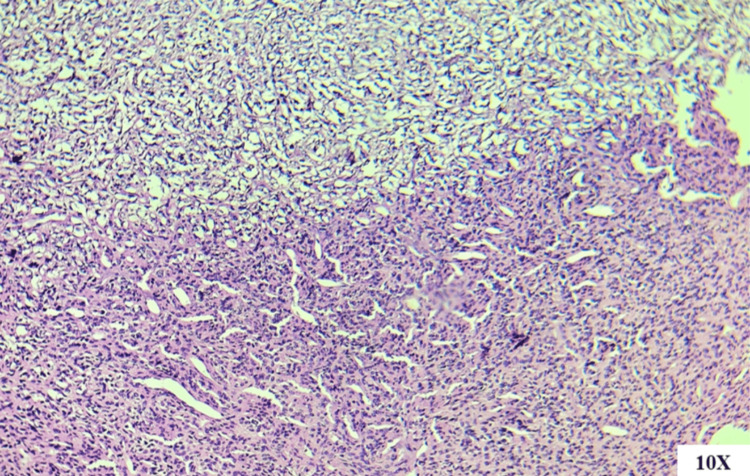
Histopathological classical pattern (hematoxylin and eosin (H&E)-stained section at 10X magnification). Histopathological section stained with H&E observed under optical microscope (10X), showing the classic pattern of hemangiopericytoid vessels in “stag horn” formation, with a transition from a hypercellular area to a hypocellular area.

In CT, the tumors were isodense with well-defined margins, marked heterogeneous enhancement, and variable edema (Figures 1-2). MRI revealed intermediate intensity on T1-weighted images and variable in T2-weighted images, with heterogeneous features. They showed avid and predominantly homogeneous enhancement. Focal diffusion restriction was observed in one-third of the cases on DWI. The edema was variable but mostly mild. The average tumor volume in supratentorial cases was 115.9 cm^3^ (range 62.3-198.5 cm^3^); infratentorial tumors measured 44.6 cm^3^ on average, and intradural spinal tumors measured 3.83 cm^3^. Of the 10 tumors, seven were classified as World Health Organization (WHO) grade 1, two as grade 2, and one as a grade 3 tumor. Figure 3 illustrates the classic histopathological patterns of these tumors.

**Figure 4:**
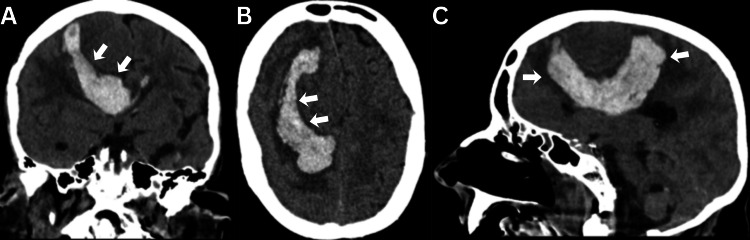
Unusual presentation with tumor hemorrhagic apoplexy. A 70-year-old woman presented with sudden neurological deterioration secondary to tumor hemorrhage. She died one month later due to a poor clinical course. This represents an unusual onset for such lesions. (A) Coronal, (B) axial, and (C) sagittal sections showing tumor dissection from the hemorrhage, clearly outlining the tumor. Arrows: Intracranial hyperdensity corresponding to peritumoral hemorrhage.

The mean number of mitoses observed was 2 per 10 high-power fields, with a range of 0-7. Necrosis was absent in 60% of the tumors (six patients); however, two tumors exhibited necrosis in 30% or more of the tissue samples. STAT6 was diffusely positive across all cases. Epithelial membrane antigen was positive in three patients. Ki67 was evaluated in seven specimens, yielding an average of 7.28% (range 2-12%). Vimentin and CD34 were assessed in seven instances, both showing five positive results (71.4% of positivity). S100 was negative in all six cases of this study. Progesterone receptors were positive in 40% of cases, with positivity ranging from 3% to 100% (Table 2). Among the 10 cases studied, one resulted in mortality (Figure 4), while the remaining patients showed no decline in the Glasgow Coma Scale, with one patient demonstrating improvement (from 14 to 15 points). Regarding the Rankin Scale, the initial (preoperative) and final (postoperative) averages were 2.7 and 1.4, respectively. On the Karnofsky Performance Scale, the average ranged from 67% to 75% (73.3% to 83.3% when excluding the fatal case) (Table 3). None of the tumors underwent initial embolization; only one was embolized prior to reoperation after resection was halted because of massive hemorrhage. Two patients received radiotherapy at doses of 50.4 and 60 Gy, respectively, administered in 30 fractions. Gross total resection was achieved in three patients during the initial surgery (30%), whereas in two others, it was accomplished during the second surgery (20%). Five patients underwent reoperation (50%). The primary intraoperative complication was massive hemorrhage, which occurred in 30% of the cases (three patients). Postoperative surgical site infection occurred in two patients (20%), and one patient (10%) experienced partial wound dehiscence. The average hospital stay was 15.25 days (range: 6-44 days). The mean follow-up period was 23.3 months (range, 1-60 months). Tumor control was achieved in 50% of patients (five cases), and no cases of metastasis were observed.

**Table 2:** Immunohistochemical profile of patients with solitary fibrous tumor. HPF: high-power field, EMA: epithelial membrane antigen, PR: progesterone receptor, NR: not reported

Case	Mitoses/10 HPF	Necrosis (%)	STAT6	EMA	Ki-67 (%)	Vimentin	CD34	S100	PR
1	2	30	+	+++	8	+++	+++	-	-
2	0	40	+	+ (focal)	10	+++	NR	NR	-
3	5	10	+	NR	12	NR	+	-	+
4	0	0	+	-	NR	-	+++	-	-
5	2	3	+	-	NR	NR	+++	NR	-
6	1	0	+	-	NR	-	+++	NR	+
7	1	0	+	-	4	NR	NR	-	+
8	2	0	+	-	10	+++	-	NR	+
9	7	0	+	+ (focal)	2	+++	NR	-	-
10	9	0	+	-	5	+++	-	-	-

**Table 3:** Clinical follow-up and outcomes. GCS: Glasgow Coma Scale, mRS: modified Rankin Scale, KPS: Karnofsky Performance Status, HPF: high-power field, EMA: epithelial membrane antigen, PR: progesterone receptor, STR: subtotal resection, GTR: gross total resection, fx: fractions, CSF: cerebrospinal fluid, NR: not reported

Case	Functional Status (GCS/mRS/KPS Pre-Post)	Embolization	Radiotherapy	Extent of Resection	Postoperative Complications	Follow-Up (Months)	Reoperation	Recurrence	Metastasis	Tumor Control
1	14-15/4-1/50-50	No	No	STR → GTR	Significant hemorrhage	42	Yes	No	No	Yes
2	15-15/1-2/90-80	Yes	60 Gy (30 fx)	STR	Significant hemorrhage	27	Yes	Yes	No	No
3	15-15/1-1/100-100	No	No	GTR	None	18	No	No	No	Yes
4	15-15/1-1/90-100	No	No	GTR	None	18	No	No	No	Yes
5	15-15/4-4/60-60	No	No	STR	Surgical site infection	13	Yes	Yes	No	No
6	6-Deceased/5-Deceased/10-Deceased	No	No	STR	Death	1	No	No	No	No
7	15-15/2-2/80-80	No	50.4 Gy (30 fx)	STR	CSF fistula; surgical site infection	30	Yes	Yes	No	No
8	15-15/2-1/70-100	No	No	STR → GTR	None	60	Yes	Yes	No	No
9	15-15/5-1/40-90	No	No	STR	Wound dehiscence	17	No	No	No	Yes
10	15-15/2-2/80-80	No	No	GTR	None	7	No	No	No	Yes

## Discussion

During the period under investigation at our center, SFTs constituted less than 1% of the central nervous system neoplasms that underwent surgical intervention. This finding aligns with the existing international literature [2,7]. As our institution primarily serves adult patients, pediatric cases were excluded; nonetheless, the age of onset was consistent with previous reports by various authors. The age range spanned from 27 to 70 years, with a mean age of 48 years, which is marginally higher than that reported by Giordan et al. Contrary to Giordan et al., who identified a higher prevalence in males, our study observed a female-to-male ratio of 7:3 [8]. In an epidemiological study by Lu et al., encompassing 650 cases, the mean age was 55 years, with no significant sex differences, and an intracranial location in 80.6% of cases, intradural spinal in 13.4%, and unspecified in 6% [7]. Alker et al. retrospectively analyzed 49 cases and reported a distribution of 54% in the supratentorial compartment, 24% in the infratentorial compartment, 14% in the spinal compartment, and 6% unspecified. Our smaller cohort exhibited a similar distribution, with 60%, 20%, and 20% of the tumors being supratentorial, infratentorial, and intradural spinal, respectively [18]. Of the two spinal tumors identified, one was located in the cervicothoracic region and the other in the lumbar region of the spine. A review encompassing 29 studies with 407 cases of both intracranial and intradural spinal schwannomas identified 109 cases (26.7%) as intradural spinal schwannomas, predominantly thoracic (45.3%), followed by cervical (32.1%) and lumbar (22.6%) [8].

The clinical presentation was nonspecific, with headache being the predominant symptom, consistent with previous findings [10]. Two patients exhibited atypical presentations, one with seizures and the other with hemorrhage. Although this condition typically involves highly vascularized lesions, spontaneous hemorrhage is uncommon. Walker et al. documented a case of a tumor located in the conus medullaris that presented with bleeding and a subacute clinical course [2,19]. Since its initial description, SFT has represented a diagnostic challenge, as it is difficult to differentiate it from meningioma, both clinically and through imaging findings [20]. Another differential diagnosis to consider is schwannoma, which was suspected in 20% of the cases in our series [8]. In imaging studies, particularly CT, SFT is nearly indistinguishable from meningiomas. Conversely, MRI has revealed certain characteristics that are useful in the differentiation between these two entities. In a systematic review, El-Abtah et al. identified a higher ADC in SFTs [21]. In our series, one-third of the cases exhibited focal diffusion pseudorestriction with elevated ADC. Another characteristic described in SFT is the absence of a dural tail sign; none of our cases presented a dual tail sign. The finding of this sign has a sensitivity of 58.6% and specificity of 94.02% for meningiomas. In addition, edema has been noted in meningiomas, particularly in certain subtypes, such as the angiomatous type. In our series, the edema was variable but with a tendency to be mild. A demographic indicator is the age at presentation, with meningiomas generally occurring at an older age than SFTs [21,22]. Tumor volume was also variable, with size proportional to the compartment of location, being largest in the supratentorial region, followed by the infratentorial region, and finally the intraspinal cases.

In our series, 70% of the cases were classified as WHO grade 1, 20% as grade 2, and 10% as grade 3. In other studies, the distribution of grades varied. Sung et al., in a retrospective study involving 60 patients using the 2016 WHO criteria, reported 6.6% grade 1, 66.6% grade 2, and 26.6% grade 3 [23]. Similarly, Fritchie et al., in a cohort of 133 patients also utilizing the 2016 WHO classification, documented 32.3% grade 1, 30.8% grade 2, and 36.8% grade 3 [24]. Macagno et al., employing the Marseille Grading System in a cohort of 132 patients, identified 55%, 38%, and 7% of patients with grade 1, grade 2, and grade 3 tumors, respectively [25]. Fritchie et al. reported an average of 2 mitoses in 10 high-power fields (range, 0-45), which is consistent with the average found in our study (range, 0-7). Regarding necrosis, they observed it in 12% of cases, whereas in our study, it was present in 40% of cases (range, 3-40%). CD34 positivity was also assessed, with Fritchie et al. finding it in 81.2% of cases; in our study, it was 71.4%, while Liu et al., in a study of 38 patients from their hospital center, reported it in 86.9% of cases [4,24]. Liu et al. reported a mean Ki-67 index value of 8.5%, which is comparable to 7.28% in our series. They also evaluated vimentin positivity, with a result of 94.7%; in our study, it was 71.4%. In Liu et al.'s study, epithelial membrane antigen was identified in 23.7% of cases, whereas our study reported a prevalence of 30%. S-100 was detected in 39.4% of their cases, but was absent in our cases [4]. Savary et al., through a retrospective observational study, reported progesterone receptor positivity in 29% of tumors, compared to 40% in our series [26]. Swaminathan et al., in a retrospective cohort study involving 34 patients who underwent microsurgery, observed a mortality rate of 29% over an average follow-up period of 79 months. In contrast, our series recorded a mortality rate of 10% with a mean follow-up of 23.3 months (ranging from 1 to 60 months). In their study, 29% of patients received preoperative embolization, whereas at our institution, only one patient underwent this procedure [27]. Lu et al. reported a mean survival of 57 months post-diagnosis, with a tumor-attributable mortality rate of 6.3%. They also noted that 42.3% of patients received radiotherapy following surgery, which is twice the 20% observed in the present study. In the same study, Lu et al. reported a gross total resection in 53.8% of the operated patients, closely aligning with the 50% gross total resection observed in our series (30% during the initial intervention) [7]. In our cases, significant hemorrhage, as documented in surgical records or exceeding 1000 mL, was reported in 30% of patients, attributed to the pronounced vascularization of the tumors. In a case series involving seven patients, Yip et al. documented an average intraoperative blood loss of 1542 mL, highlighting the hemorrhagic potential of these lesions [3]. Although distant metastases have been reported relatively frequently, Lu et al. documented an incidence of approximately 3.1% in their population-based study [7]. In our series, no evidence of dissemination was observed, and 50% of the patients achieved adequate control with no recurrence during follow-up, which has been associated with a favorable prognosis [10].

This study's limitations include its retrospective observational nature, the low incidence of these tumors, which translates to a low sample size (10 patients), a single center (although referral hospital) experience that may predispose to selection bias, and the relatively short follow-up period that may impede the generalization of the results.

## Conclusions

The rare occurrence of these tumors makes clinical suspicion difficult, with no pathognomonic clinical signs or radiographic features for diagnosis. An SFT may be considered when imaging shows a vascularized extra-axial dural-based lesion without the "dural tail" sign and high ADC values on MRI. Considering this, differential diagnosis may improve preoperative planning (embolization) by anticipating hemorrhagic complications. Limited diagnostic testing may contribute to underreporting of SFTs. This case series represents Mexico's largest documented record of SFT in the central nervous system, with findings consistent with the international literature, although more Latin American research is needed. Generalizability is limited by the study's features.

## References

[1] Klemperer P, Coleman BR (1992). Primary neoplasms of the pleura. A report of five cases. Am J Ind Med.

[2] WHO Classification of Tumours Editorial Board (2021). Central Nervous System Tumors. https://publications.iarc.who.int/Book-And-Report-Series/Who-Classification-Of-Tumours/Central-Nervous-System-Tumours-2021.

[3] Yip CM, Hsu SS, Liao WC (2020). Intracranial solitary fibrous tumor/hemangiopericytoma - a case series. Surg Neurol Int.

[4] Liu J, Wu S, Zhao K, Wang J, Shu K, Lei T (2022). Clinical features, management, and prognostic factors of intracranial solitary fibrous tumor. Front Oncol.

[5] Al Armashi AR, Alkrekshi A, Al Zubaidi A (2022). Grade III solitary fibrous tumor/hemangiopericytoma: an enthralling intracranial tumor-a case report and literature review. Radiol Case Rep.

[6] Janik AM, Terlecka A, Spałek MJ (2023). Diagnostics and treatment of extrameningeal solitary fibrous tumors. Cancers (Basel).

[7] Lu T, Xu H, Dong X, Jin Z, Wang Y (2022). Epidemiology and survival of patients with central nervous system solitary fibrous tumors: a population-based analysis. Front Oncol.

[8] Giordan E, Marton E, Wennberg AM, Guerriero A, Canova G (2021). A review of solitary fibrous tumor/hemangiopericytoma tumor and a comparison of risk factors for recurrence, metastases, and death among patients with spinal and intracranial tumors. Neurosurg Rev.

[9] Claus E, Seynaeve P, Ceuppens J, Vanneste A, Verstraete K (2017). Intracranial solitary fibrous tumor. J Belg Soc Radiol.

[10] Gubian A, Ganau M, Cebula H (2019). Intracranial solitary fibrous tumors: a heterogeneous entity with an uncertain clinical behavior. World Neurosurg.

[11] Chen T, Jiang B, Zheng Y, She D, Zhang H, Xing Z, Cao D (2020). Differentiating intracranial solitary fibrous tumor/hemangiopericytoma from meningioma using diffusion-weighted imaging and susceptibility-weighted imaging. Neuroradiology.

[12] Clarençon F, Bonneville F, Rousseau A (2011). Intracranial solitary fibrous tumor: imaging findings. Eur J Radiol.

[13] Gopakumar S, Srinivasan VM, Hadley CC (2021). Intracranial solitary fibrous tumor of the skull base: 2 cases and systematic review of the literature. World Neurosurg.

[14] Tariq MU, Din NU, Abdul-Ghafar J, Park YK (2021). The many faces of solitary fibrous tumor; diversity of histological features, differential diagnosis and role of molecular studies and surrogate markers in avoiding misdiagnosis and predicting the behavior. Diagn Pathol.

[15] Piscopo AJ, Chowdhury AJ, Teferi N (2024). Surgical management of craniospinal axis solitary fibrous tumors: a single-institution case series and comprehensive review of the literature. Neurosurgery.

[16] Cohen-Inbar O, Lee CC, Mousavi SH (2017). Stereotactic radiosurgery for intracranial hemangiopericytomas: a multicenter study. J Neurosurg.

[17] Golub D, McBriar JD, Donaldson H (2023). Postoperative stereotactic radiosurgery for intracranial solitary fibrous tumors: systematic review and pooled quantitative analysis. J Neurooncol.

[18] Alker JP, Rahmanzade R, Held T (2025). High-grade, metastatic disease, and adjuvant radiotherapy are independent prognostic factors for progression-free survival in patients with solitary fibrous tumors. Neurooncol Adv.

[19] Walker CT, Amene CS, Pannell JS, Santiago-Dieppa DR, Rennert RC, Hansen LA, Khalessi AA (2015). Hemorrhagic intramedullary solitary fibrous tumor of the conus medullaris: case report. J Neurosurg Spine.

[20] Carneiro SS, Scheithauer BW, Nascimento AG, Hirose T, Davis DH (1996). Solitary fibrous tumor of the meninges: a lesion distinct from fibrous meningioma. A clinicopathologic and immunohistochemical study. Am J Clin Pathol.

[21] El-Abtah ME, Murayi R, Lee J, Recinos PF, Kshettry VR (2023). Radiological differentiation between intracranial meningioma and solitary fibrous tumor/hemangiopericytoma: a systematic literature review. World Neurosurg.

[22] Cordia QC, Dijkstra BM, Groen RJ (2025). The dural tail in intracranial meningioma: heads up or tail down? A systematic review of the literature. Neurosurg Rev.

[23] Sung KS, Moon JH, Kim EH (2019). Solitary fibrous tumor/hemangiopericytoma: treatment results based on the 2016 WHO classification. J Neurosurg.

[24] Fritchie K, Jensch K, Moskalev EA (2019). The impact of histopathology and NAB2-STAT6 fusion subtype in classification and grading of meningeal solitary fibrous tumor/hemangiopericytoma. Acta Neuropathol.

[25] Macagno N, Vogels R, Appay R (2019). Grading of meningeal solitary fibrous tumors/hemangiopericytomas: analysis of the prognostic value of the Marseille Grading System in a cohort of 132 patients. Brain Pathol.

[26] Savary C, Rousselet MC, Michalak S, Fournier HD, Taris M, Loussouarn D, Rousseau A (2016). Solitary fibrous tumors and hemangiopericytomas of the meninges: Immunophenotype and histoprognosis in a series of 17 cases [Article in French]. Ann Pathol.

[27] Swaminathan S, Ruzevick J, Venur V (2022). Intracranial solitary fibrous tumor/hemangiopericytoma treated with microsurgical resection: retrospective cohort analysis of a single-center experience. Ther Clin Risk Manag.

